# Enzymatic Debriding Agents Are Safe in Wounds With High Bacterial Bioburdens and Stimulate Healing

**Published:** 2008-04-07

**Authors:** Wyatt G. Payne, R. Emerick Salas, Francis Ko, Deepak K. Naidu, Guillermo Donate, Terry E. Wright, Martin C. Robson

**Affiliations:** From the Institute for Tissue Regeneration, Repair, and Rehabilitation, Bay Pines VA Medical Center, Bay Pines, Fla; and Division of Plastic Surgery, University of South Florida Tampa.

## Abstract

**Background:** Debridement is essential for successful wound management. Enzymatic debridement is commonly utilized in wound care but has been reported to be unsafe in wounds with significant bacterial bioburden, unless used in conjunction with topical antimicrobials. We examine this hypothesis with 2 commercially available, commonly used preparations of enzymatic debriding agents. **Materials and Methods:** Using a standard rodent model of a chronically infected granulating wound with bacterial levels greater than 1 × 10^5^ Colony Forming Units per gram of tissue, commercially available preparations of collagenase and papain-urea were utilized to investigate the response of infected wounds to these preparations, and to evaluate their ability to overcome the inhibition of infection on wound healing. Quantitative bacteriology of tissue biopsies and wound healing trajectories were used to compare the preparations to saline-treated negative controls. **Results:** Collagenase- and papain-urea-treated wounds demonstrated a reduction in bacterial burden of wounds to < 10^5^ colony forming units/gram of tissue (*P* < .05). This decrease in bacterial bioburden occurred rapidly, allowing wounds to achieve bacterial balance in a short period of time. Wounds treated with enzymatic debriding agents healed significantly faster and to greater extent than saline-treated controls (*P* < .01); a direct reflection of the decreasing bacterial load of the wound. **Conclusions:** Collagenase and papain-urea appear beneficial and safe even in wounds with high bacterial loads, and appear to significantly aid extent and rate of healing, probably by lowering bacterial burden through their positive enzymatic actions on bacteria and necrotic tissue.

Debridement is one of the essential tools of wound management.^1^ It is defined as the removal of nonviable material, foreign bodies, and poorly healing tissue from a wound, and it facilitates the processes of granulation, contraction, epithelialization, and healing. The most direct form of debridement is surgical excision, although other reasonable options exist for patients who are poor surgical candidates or who have wounds in need of less aggressive debridement. These alternative debridement options include the following: mechanical debridement, which is exemplified by wet to dry dressings or pressure irrigation; autolytic debridement, in which occlusive dressings allow wound proteases to liquefy necrotic tissue; biologic debridement, which utilizes maggot therapy; and enzymatic debridement, which utilizes agents such as collagenase or papain-urea. Collagenase has been shown to be useful for degredation of collagen and elastin but not fibrin. Papain-urea's main action is to solubilize fibrin.

Numerous enzyme preparations have been investigated and used clinically since the 1940s.^2^ It has been reported that proteolytic enzyme treatment of wounds with substantial bacteria bioburden is unsafe unless treated in conjunction with topical antimicrobials. These enzymatic agents demonstrated rapid eschar degradation but allowed for significant bacterial proliferation and invasion.^5^–^5^ These reports were based on studies indicating that enzyme-treated wounds were associated with increased bacterial counts as well as studies indicating that topical enzyme treatment of burn wounds was associated with the development of burn wound sepsis. The potential for systemic sepsis could be reduced by concomitant use of topical antimicrobial agents along with the topical enzyme treatment regimen.^2^,^3^ Collagenase and papain-urea are frequently utilized, when indicated, for chronic wound care, yet the effects of enzymatic debriding agents on the microbiology of chronic-infected wounds has not been extensively characterized. Wound preparation methods are necessary to manage wound healing in an effort to accelerate healing and allow for complete closure secondarily, or to prepare for surgical closure.^6^ Frequent sharp debridement has been shown to accelerate wound healing and it has been postulated that enzymatic wound debridement may therapeutically benefit wound healing in a similar way.^1^,^6^ This study evaluates the microbiological effects of the actions of collagenase or papain-urea in a chronically infected wound model.

## METHODS

A standard rodent model of a chronically infected granulating wound was utilized.^7^–^10^ All animal experiments were approved by the Bay Pines VAMC Animal Care and Use Committee. Fifteen anesthetized male Sprague-Dawley rats weighing 300–350 g underwent a 30-cm^2^ full-thickness dorsal scald burn produced by immersion in boiling water for 15 seconds. The wounds were allowed to cool and then were inoculated with 5 × 10^9^ *E coli* (ATTC #25922; American Type Tissue Culture, Rockville, Maryland), to produce an infected wound. *E coli* was utilized because of its relatively high frequency of isolation in wounds, its ability to sustain elevated levels of greater than 1 × 10^5^ Colony Forming Units per gram of tissue, and its previously noted utility in this model to emulate a human chronically infected burn wound.^7^ On postwounding day 5, the wound eschar was excised, resulting in a chronically infected granulating wound with greater than 10^8^ bacteria per gram of tissue. The animals were divided into 3 groups of 5. Group I wounds were treated daily with isotonic sodium chloride solution (control). Group II wounds were treated daily with an application of commercially available collagenase preparation. Group III wounds were treated daily with a commercially available preparation of papain-urea. Data were collected twice per week. Wounds areas were evaluated by serially tracing wound circumferences and performing digital planimetry (Sigma Scan Jandel Scientific, Corte Madera, California). For each animal's wound area data, a Gompertz equation was fitted. Comparison among groups was performed using SAS (SAS/STAT Guide for Personal Computer, Version 6 Manual, Los Angeles, BMDP Statistical Software, Inc, 1988).^11^,^12^ Quantitative bacteriology was performed serially by tissue biopsy samples, in previously described standardized fashion.^13^

## RESULTS

**Figure 1:**
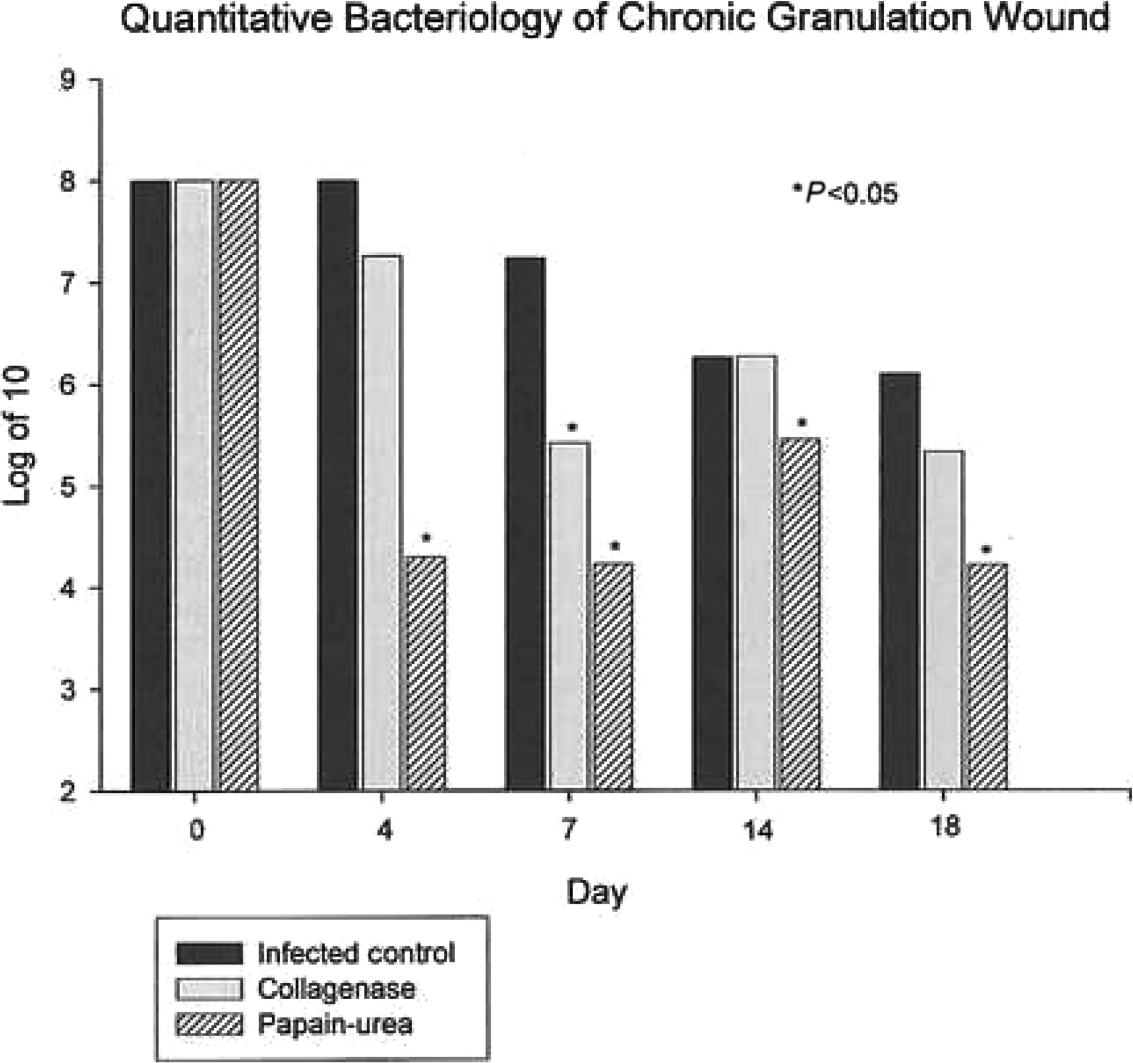
Quantative bacteriology of infected chronic granulating wounds treated with isotonic sodium chloride solution (control), collagenase, and papain-urea. Decreasing bacterial counts (Colony Forming Units per gram of tissue) overtime indicate significantly diminished bacterial burden in wounds treated with papain-urea.

Quantitative bacteriology demonstrated a reduction of tissue bacterial levels, which was significantly greater in enzyme-treated wounds. Wound bacterial counts decreased rapidly win the groups treated with enzyme debriding agents. Papain-urea reduced tissue bacterial levels to <10^5^ per gram of tissue (*P* < .05), and to a greater extent than isotonic sodium chloride solution controls or collagenase (Fig 1).

**Figure 2:**
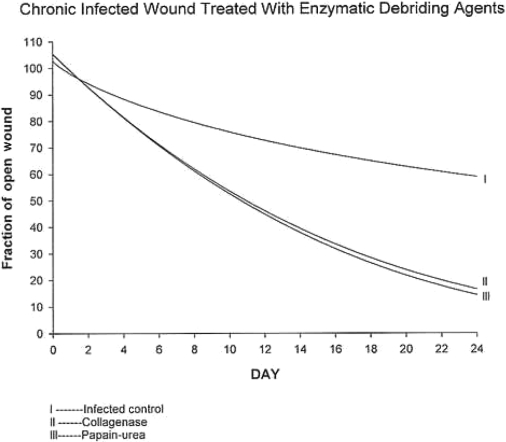
Wound closure rates for chronic granulating wounds treated with isotonic sodium chloride solution (control), collagenase, and papain-urea. Statistically significant accelerated closure rates and extents for wounds treated with collagenase or papain-urea versus saline control.

Wound closure rates were significantly accelerated in groups treated with collagenase or papain-urea as compared to saline-treated controls (*P* < .05) (Fig 2). Closure rate parallels the rapid decrease in bacterial load and may be a direct result of this effect.

## DISCUSSION

Debridement is an integral component for care of wounds of all etiologies that contain necrotic tissue, high bacterial burden, or other complicating unwanted elements.^1^ Surgical (sharp) debridement is the most rapid, direct, and effective methods of debridement. However, not all wounds or patients are candidates for surgery. Nonsurgical enzymatic debridment presents an alternative to sharp surgical debridement, can be accomplished with bedside dressing change regimens, and is available to nonsurgeon practitioners.^2^–^6^

Enzymatic debriding agents have long been used in burn wound treatment regimens due to their effectiveness and ease of use.^3^–^5^ However, data would indicate cautious approach when utilizing enzymatic agents in burn wounds with significant bacterial bioburdens.^2^–^5^ There are concerns that enzymatic debridement in the face of tissue bacterial loads greater than 10^5^ may predispose to worsening invasive infection, leading to sepsis.^2^–^4^ Concomitant topical antimicrobials were recommended in an attempt to avoid this complication.^3^ This current experiment evaluates 2 of the most commonly used enzymatic debriding agents, collagenase and papain-urea, and give evidence that these agents appear to be beneficial and safe for use in a chronically infected wound model, even in the face of significant bioburden.

Wound care regimens that are simple and effective have greater compliance and success. Simplification of wound care by (safely) reducing the number of preparations used for care may help improve wound-healing outcomes.

Commercially available collagenase and papain-urea enzymatic debriding agents are commonly used for wound care and show effectiveness in wound bed preparation. As compared with previous proteolytic enzyme preparations, both collagenase and papain-urea appear to reduce bacterial load to a level of 10^5^ or fewer bacteria per gram of tissue; a level of bacterial burden consistent with normal wound healing (Fig 1). Wounds treated with collagenase or papain-urea close at an accelerated rate when compared with wounds treated with isotonic sodium chloride solution (Fig 2). Although this effect is due in part to reduction of bacterial burden, some of this accelerated wound healing may be due to other wound healing properties of these specific enzymatic preparations. It appears that because of their antimicrobial properties and wound healing effectiveness, collagenase and papain-urea may be safely utilized without concomitant topical antimicrobials in chronic-infected wounds.

## CONCLUSION

Concerns of invasive infection after treatment of burn wounds by enzymatic debridement have existed for quite some time. Previous experimental and clinical data had substantiated these concerns and resulted in recommendations to use concomitant topical antimicrobials to avoid this complication. In this current experiment, 2 commonly used enzymatic debriding agents, collagenase and papain-urea, appear safe and beneficial in wounds with high tissue levels of bacteria. These agents demonstrate an ability to reduce wound bioburden as well as promote wound healing. Wounds treated with these enzymatic debriding agents appear safe to use without concomitant topical antimicrobial therapy.
